# Development of ovarian tumour causes significant loss of muscle and adipose tissue: a novel mouse model for cancer cachexia study

**DOI:** 10.1002/jcsm.12864

**Published:** 2022-01-19

**Authors:** Yi Luan, Yaqi Zhang, Seok‐Yeong Yu, Mikyoung You, Pauline C. Xu, Soonkyu Chung, Takeshi Kurita, Jie Zhu, So‐Youn Kim

**Affiliations:** ^1^ Olson Center for Women's Health, Department of Obstetrics and Gynecology, Fred & Pamela Buffett Cancer Center, College of Medicine University of Nebraska Medical Center Omaha NE USA; ^2^ Division of Reproductive Science in Medicine, Department of Obstetrics and Gynecology, Feinberg School of Medicine Northwestern University Chicago IL USA; ^3^ Department of Nutrition, School of Public Health and Health Sciences University of Massachusetts Amherst Amherst MA USA; ^4^ Department of Cancer Biology & Genetics, The Comprehensive Cancer Center Ohio State University Columbus OH USA

**Keywords:** Cancer cachexia, Activin A, Muscle, Adipose, Ovarian tumour, Mouse model

## Abstract

**Background:**

Cancer‐associated cachexia (CAC) is a complex syndrome of progressive muscle wasting and adipose loss with metabolic dysfunction, severely increasing the morbidity and mortality risk in cancer patients. However, there are limited studies focused on the underlying mechanisms of the progression of CAC due to the complexity of this syndrome and the lack of preclinical models that mimics its stagewise progression.

**Methods:**

We characterized the initiation and progression of CAC in transgenic female mice with ovarian tumours. We measured proposed CAC biomarkers (activin A, GDF15, IL‐6, IL‐1β, and TNF‐α) in sera (*n* = 6) of this mouse model. The changes of activin A and GDF15 (*n* = 6) were correlated with the decline of bodyweight over time. Morphometry and signalling markers of muscle atrophy (*n* ≥ 6) and adipose tissue wasting (*n* ≥ 7) were assessed during CAC progression.

**Results:**

Cancer‐associated cachexia symptoms of the transgenic mice model used in this study mimic the progression of CAC seen in humans, including drastic body weight loss, skeletal muscle atrophy, and adipose tissue wasting. Serum levels of two cachexia biomarkers, activin A and GDF15, increased significantly during cachexia progression (76‐folds and 10‐folds, respectively). Overactivation of proteolytic activity was detected in skeletal muscle through up‐regulating muscle‐specific E3 ligases *Atrogin‐1* and *Murf‐1* (16‐folds and 14‐folds, respectively) with decreasing cross‐sectional area of muscle fibres (*P* < 0.001). Muscle wasting mechanisms related with p‐p38 MAPK, FOXO3, and p‐AMPKα were highly activated in concurrence with an elevation in serum activin A. Dramatic fat loss was also observed in this mouse model with decreased fat mass (*n* ≥ 6) and white adipocytes sizes (*n* = 6) (*P* < 0.0001). The adipose tissue wasting was based on thermogenesis, supported by the up‐regulation of uncoupling protein 1 (UCP1). Fibrosis in adipose tissue was also observed in concurrence with adipose tissue loss (*n* ≥ 13) (*p* < 0.0001).

**Conclusions:**

Our novel preclinical CAC mouse model mimics human CAC phenotypes and serum biomarkers. The mouse model in this study showed proteolysis in muscle atrophy, browning in adipose tissue wasting, elevation of serum activin A and GDF15, and atrophy of pancreas and liver. This mouse line would be the best preclinical model to aid in clarifying molecular mediators of CAC and dissecting metabolic dysfunction and tissue atrophy during the progression of CAC.

## Introduction

Cachexia is a life‐threatening metabolic syndrome in advanced cancer patients that severely impairs physical function, increases the severity of treatment‐related toxicity, and accounts for nearly 25% of cancer‐related deaths.^1^ Cancer‐associated cachexia (CAC) is characterized by progressive weight loss over time, most commonly with ≥5% weight loss in 6 months.^2^ Evidence suggests that early treatment of CAC is critical, as weight loss >5% is associated with higher mortality risk,^3^ and reversing CAC is also more challenging once patients have reached advanced stages of disease.

Most knowledge regarding the mechanisms of CAC and possible therapeutic approaches is derived from preclinical animal models, including syngeneic cancer cell graft models, xenograft tumour models, genetically engineered spontaneous tumour models, and carcinogen‐induced cancer models.^4^ However, a major drawback of these models is the relatively short period between onset of CAC symptoms and death due to aggressive growth of non‐spontaneous cancers. Human tumour xenograft models can closely mimic human tumour characteristics; however, the application of such models to the study of cachexia is limited since immune‐deficient recipient animals lack the inflammatory response critical for development of CAC.^5^ Establishment of good preclinical models that develop tumours spontaneously and recapitulate tumour–host interactions is needed to better understand the mechanisms of CAC development and for use in drug testing.

Muscle wasting is one of the hallmarks of CAC and is associated with increased morbidity and mortality; prevention of muscle wasting has been shown to increase survival in tumour‐bearing animal models.^6^, ^7^ Previous studies have described muscle wasting in CAC as dysregulation of homeostasis between anabolic and catabolic processes in skeletal muscle,^8^ with the balance tipped towards a catabolic state as a result of activated proteolytic systems. Apart from muscle wasting, loss of adipose tissue is another important component of CAC. White adipose tissue (WAT) is known as an energy storage organ, while brown adipose tissue (BAT) is responsible for heat production.^9^ Brown‐like adipocytes accumulate within WAT by a process termed WAT “browning” which leads to elevated thermogenic energy expenditure and contributes to negative energy balance.^10^ The brown‐like adipocytes, so called ‘beige’ adipocytes, express uncoupling protein 1 (UCP1) to dissipate the mitochondrial energy into heat.^11^, ^12^ Clinical analysis has shown that adipose tissue wasting results from a reduction in fat mass owing to lipolysis and browning rather than apoptosis of adipocytes.^13^ Inhibition of WAT browning has been proposed as a therapeutic potential to ameliorate CAC progression in cancer patients.^14^

Although the development of CAC is correlated with increased circulating inflammatory factors derived from host or tumour cells such as interleukin (IL)‐6^13^, ^15^ and tumour necrosis factor‐alpha (TNF‐α),^16^ inhibiting the inflammatory response is ineffective in ameliorating cachexia in patients. TGF‐β superfamily members such as activin A,^17^, ^18^ myostatin,^19^ and GDF15^20^ have been shown to affect CAC development by negatively regulating the balance between the synthesis and degradation of muscle proteins. The soluble ActRIIB‐Fc (activin receptor type IIB), which blocks activin and myostatin signalling, has been shown to completely reverse muscle wasting and weight loss in CAC mouse models.^5^ Antibodies and small molecules that target activin have also been developed.^21^, ^22^ In a Phase 2 clinical trial, Bimagrumab, a human monoclonal antibody that blocks activin Type II receptors, has been shown to increase skeletal muscle mass^23^ without updated outcomes regarding cachectic fat loss.

In the present study, we established a novel mouse model that shows progressive CAC as tumours grow. We characterized morphological alterations, hormone profiles, and wasting in muscle and adipose tissue with key molecules along with CAC development. In all, we found that our novel preclinical CAC mouse model mimics human cancer cachexia progression and is a potentially important tool for studying the underlying mechanism of tissue loss and metabolic alterations during the progression of cancer cachexia.

## Materials and methods

### Animals

Transgenic mice with oocyte‐specific expression of constitutively active PI3K (Pik3ca*) were generated as previously described.^24^ Homozygous female with a Cre‐inducible knock‐in allele for Pik3ca* were crossed with heterozygous *Gdf9‐iCre* ^***+/−***^ male. Cre− or Cre+ female mice were determined by the presence of *Gdf9‐iCre* transgene (*Figure* S1A). Cre− littermates were used as controls. All procedures involving mice were approved by the Institutional Animal Care and Use Committee (IACUC) at University of Nebraska Medical Center (UNMC). Animals were provided with food and water *ad libitum* and kept in Comparative Medicine facilities (UNMC, Omaha, NE, USA). Temperature, humidity, and photoperiod (10/14 photoperiod) were kept constant. Food intake was measured for 3 days before being sacrificed and calculated by food intake per mouse per day (g).

### Body mass composition

Dual X‐ray absorptiometry (DEXA) imaging was performed to measure mouse body composition. DEXA imaging was taken before a day of euthanasia at postnatal day (PD) 64 and PD82. Animals were lightly anaesthetised with isoflurane and imaged with Piximus I (Inside/Outside, Fitchburg, WI, USA). Vendor‐supplied software (Piximus I, GE Lunar) was used to identify regions of interest (ROIs) encompassing the mouse chest/abdomen/pelvis for determination of bone mass density, bone mineral content, bone area, tissue area, ratio of soft‐tissue attenuation, total tissue mass, and per cent adiposity (% fat). Data were analysed by repeated‐measures analysis of variance (ANOVA, implemented in MATLAB 2011b) with genotype and mouse age.

### Enzyme‐linked immunosorbent assay

Blood was collected by cardiac puncture. Serum activin A and GDF15 levels were determined by enzyme‐linked immunosorbent assay (ELISA) kits of activin A (AL‐110, Ansh Labs, Webster, TX, USA) and GDF15 (MGD150, R&D Systems, Minneapolis, MN, USA). The limits of detection for these kits were 0.065 ng/mL and 7.8 pg/mL, respectively. Serum IL‐6, IL‐1β, and TNF‐α levels were measured using ELISA kits (BMS603‐2 for IL‐6, BMS600‐2 for IL‐1β, and BMS607‐3 for TNF‐α from Invitrogen, Carlsbad, CA, USA) with sensitivities of 6.5, 1.2, and 3.7 pg/mL, respectively.

### Measurement of body composition and thermal release

The measurement of lean body mass and fat mass were conducted by Quantitative Nuclear Magnetic Resonance (qNMR; EchoMRI™, Houston, TX, USA) analysis. Whole‐body magnetic resonance imaging (MRI) measurements were conducted using a dedicated high‐field (7Tesla) Bruker 7T ClinScan MRI system to detect tumour growth and assessing body composition and distribution of fat tissue. To detect thermal release, an infrared (IR) camera (A655sc, FLIR Systems Inc., Wilsonville, OR, USA) was used to capture images of the surface body temperature. FLIR Research IR program software was used to display surface heat release via a colour palette representing temperatures between 25°C and 40°C.

### Histology, immunofluorescence, and immunohistochemistry assays

The tibialis anterior (TA), gastrocnemius, and quadriceps muscles; gonadal, inguinal, and brown adipose tissue; liver; and stomach were collected from mice at PD65 and PD83 and fixed in Modified Davidson's Fixative (64,133–50, Electron Microscopy Sciences, PA, USA) for 24 h at 4°C. Tissues were then processed, embedded in paraffin, and sectioned with 5 μm thickness. Haematoxylin and eosin (H&E) staining was performed using standard methods. TA, gastrocnemius, and quadriceps muscles were embedded in Tissue‐Tek* O.C.T. Compound (4583, Sakura Finetek USA, Inc., Torrance, CA, USA), frozen on dry ice and sectioned with 10 μm thickness and stored at −80°C. All images were taken with the EVOS M7000 Imaging System (AMF700, Invitrogen, Carlsbad, CA, USA). TA muscle paraffin‐embedded sections were stained with anti‐laminin antibody (L9393, 1:100, Sigma‐Aldrich, St. Louis, MO, USA) for myofibre cross‐sectional area (CSA) and quantified using ImageJ (NIH software version 1.50i). Muscle cryosections were stained with anti‐myosin heavy chain (MHC) I (BA‐F8, 1:50, Developmental Studies Hybridoma Bank, Iowa City, IA) and MHCIIA (SC‐71, 1:50, DSHB, Iowa City, IA). Fibre CSA measurements for MHCI and MHCIIA were performed by outlining all fibres within a muscle cross‐section and quantified using ImageJ. Adipose tissue sections were stained with anti‐UCP1 antibody (ab10983, 1:50, Abcam, Cambridge, MA, USA).

### Adipocyte size measurement

Adipocyte size was measured by analysing images of H&E‐stained adipose tissue sections using Adiposoft 1.14 in Manual Model. Three adipose tissue sections per mouse were analysed. About 300 adipocytes per mouse were measured for an average diameter.

### Picro‐sirius red staining

To visualize collagen I and III fibres, adipose tissue sections were stained using the Picro‐Sirius Red Stain Kit (MER PSR1, Mercedes Scientific, Lakewood Ranch, FL, USA).

### Immunoblot analysis

Muscle tissues were homogenized in lysis buffer with protease inhibitor (04693116001, Roche Life Science, Indianapolis, IN, USA) and phosphatase inhibitor cocktails (P0044, MilliporeSigma, Burlington, MA, USA). Proteins (30 μg) measured by the BCA protein assay kit (23225, Thermo Fisher Scientific, Waltham, MA, USA) were loaded into a NuPAGE 4–12% gradient Bis‐Tris gel (NP0321PK2, Thermo Fisher Scientific) and transferred to a nitrocellulose membrane using an iBlot™ 2 Gel Transfer Device (IB21001, Thermo Fisher Scientific). The blots were probed with primary antibodies followed by secondary antibodies (Supporting information). Proteins were detected by Luminata Crescendo immunoblot HRP substrate (WBLUR0500, MilliporeSigma) and exposed using a c500 imaging system (Azure Biosystems, Dublin, CA, USA). Protein quantification was performed by densitometry analysis on ImageJ.

### Real‐time quantitative PCR

Total RNA was collected using the RNeasy Lipid Tissue Mini Kit (74804, Qiagen Inc.) for adipose tissues and the RNeasy Fibrous Tissue Mini Kit (74704, Qiagen Inc.) for TA muscles. RNA samples were reverse transcribed to cDNA by Superscript III First‐Strand Synthesis Supermix (18080‐400, Thermo Fisher Scientific) or the High‐Capacity RNA‐to‐cDNA™ Kit (4387406, Applied Biosystems, Foster City, CA, USA). TaqMan® probes are listed in Supporting information. The experiment was performed in triplicates using a StepOnePlus real‐time PCR system (4376600, Applied Biosystems).

### Statistical analysis

Graphs were generated by Prism 9.1.1 software (GraphPad Software Version 9, Inc., CA, USA) and were presented as mean with ±S.E.M. Unpaired two‐tailed Student's *t* tests was used for the comparison of means between two datasets. One‐way ANOVA with Tukey's post hoc test was used for comparison of the means among more than three datasets. *P* values of less than 0.05 were considered statistically significant. Data are shown as mean with ±S.E.M. of biological replicates. n.s. represents not significant, and *, **, ***, and **** represent *P* < 0.05, *P* < 0.01, *P* < 0.001, and *P* < 0.0001, respectively.

## Results

### Pik3ca* mice develop systemic cancer cachexia‐like symptoms

**Figure 1:**
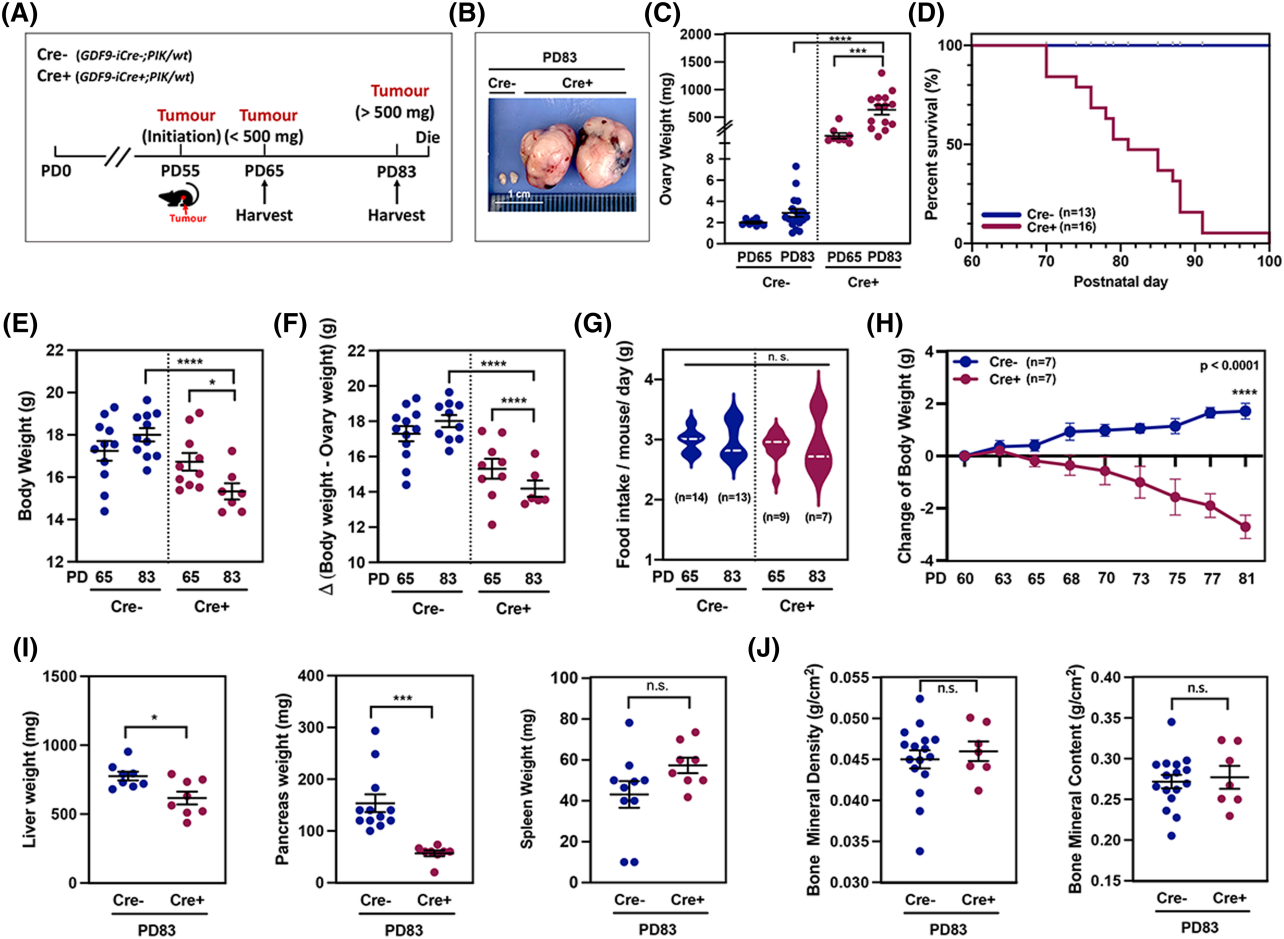
Cre+ mouse develops systemic cancer‐associated cachexia (CAC)‐like symptoms. (*A*) Schematic of tumour development in Cre+ mice. (*B*) Ovarian tumours in Cre+ and normal ovary in Cre− mice. (*C*) Ovary weight of Cre− mice on PD65 (*n* = 8) and PD83 (*n* = 18) and Cre+ mice on PD65 (*n* = 8) and PD83 (*n* = 14). (*D*) Percentage of survival of Cre+ and Cre− mice. (*E*) Bodyweight of Cre− mice on PD65 (*n* = 11) and PD83 (*n* = 11), and Cre+ mice on PD65 (*n* = 10) and PD83 (*n* = 7). (*F*) Bodyweight minus ovary weight of Cre− mice on PD65 (*n* = 12) and PD83 (*n* = 10) and Cre+ mice on PD65 (*n* = 9) and PD83 (*n* = 6). (*G*) Food intake/mouse/day of Cre− and Cre+ mice. (*H*) Change in bodyweight of Cre+ and Cre− mice. (*I*) Weight of liver (*n* = 8 for Cre− and *n* = 8 for Cre+), pancreas (*n* = 12 for Cre− and *n* = 8 for Cre+), and spleen (*n* = 10 for Cre− and *n* = 8 for Cre+). (*J*) Bone mineral density and content measured by dual X‐ray absorptiometry scan of Cre− (*n* = 16) and Cre+ (*n* = 7) mice.

Cre+ female mice initiated tumorigenesis at around PD55 and developed bilateral ovarian tumours at PD83 (*Figure* 1A and 1B) with a hunched posture and elongated incisors at PD83 (*Figure* S1B and S1C). Along with tumour formation and growth (*Figures* 1C, S1D, and S1E), Cre+ mice died between PD70 and PD100 (*Figure* 1D). Cre+ mice at PD83 showed significantly lower body weight than that of PD65 groups even with large tumours (*Figure* 1E). To examine the difference in net body weight, body weight minus tumour weight was compared. Body weight dramatically declined from PD65 to PD83 (*Figure* 1F), while there was no significant difference in food intake between Cre− and Cre+ mice (*Figure* 1G). Although the Cre+ mice displayed significant growth of ovarian tumours, their bodyweight decreased dramatically when compared with the control mice (*Figure* 1H). Cre+ mice at PD83 displayed additional abnormalities in various organs including decreased size and weight of liver and pancreas, pronounced cell apoptosis around the portal vein, depleted stomach columnar epithelium, and disorganized gastric glands (*Figures* 1I and S2B). However, there was no significant depletion in spleen, bone and tissue areas, and total tissue mass, showing similar bone mineral density and content measured by DEXA scan (*Figures* 1I, 1J, and S2C). The density of soft tissue showed a significant increase in Cre+ mice at PD83 due to the development of ovarian tumours (*Figure* S2D).

### Cre+ mice have elevated serum cachectic biomarkers during the progression of cancer cachexia

**Figure 2:**
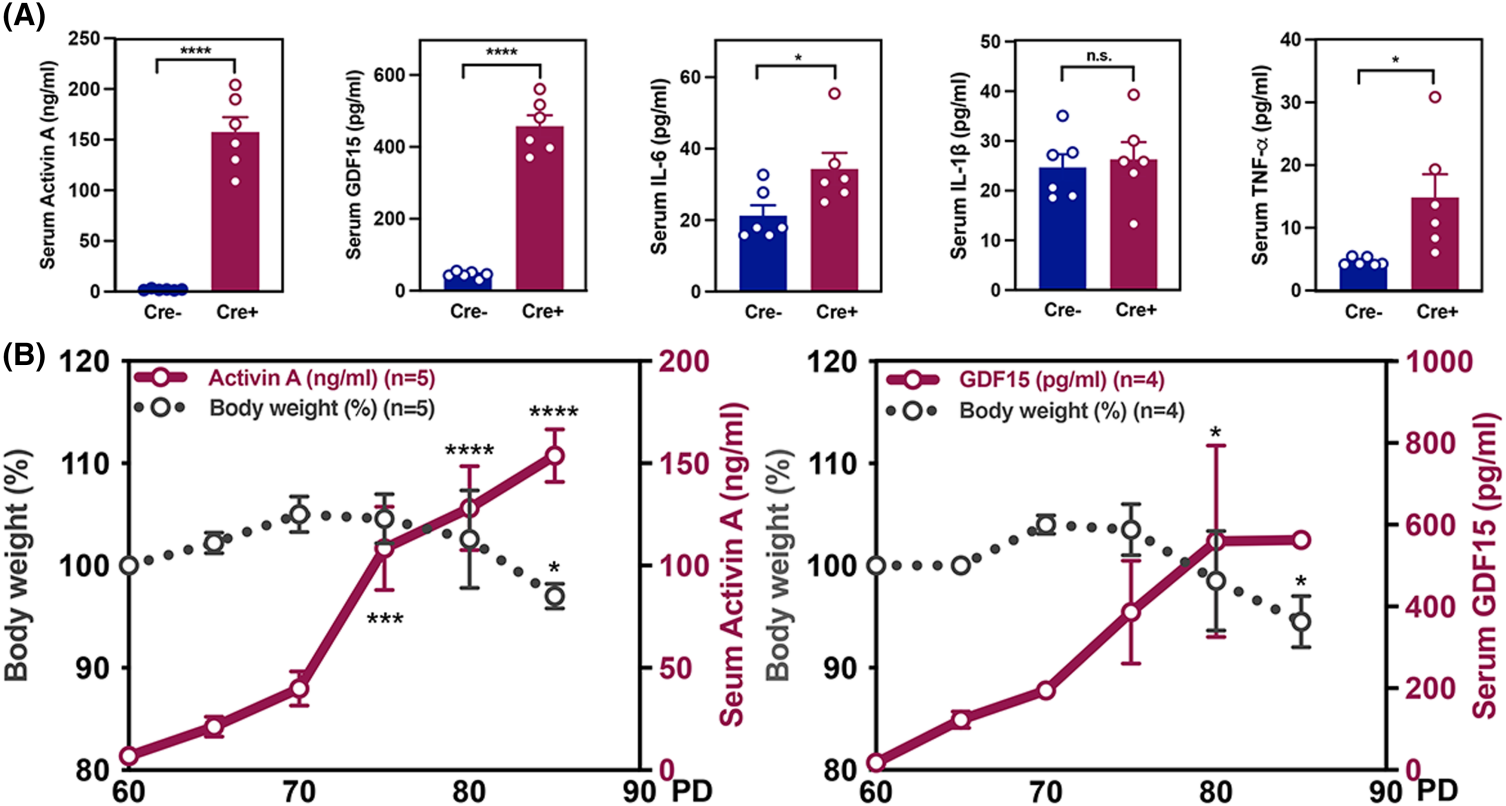
Weight loss is correlated with serum levels of activin A and GDF15. (*A*) Serum levels of activin A, GDF15, IL‐6, IL‐1β, and TNF‐α in Cre+ (*n* = 6) and Cre− mice (*n* = 6) at PD83. (*B*) Inverse correlation of body weight changes with serum activin A and GDF15 in Cre+ mice (*n* = 4).

Skeletal muscle and adipose tissue wasting in CAC is associated with an increase in various factors that derive from host or tumour cells to ascertain key factors in our cachectic mouse model, serum activin A, GDF15, and other inflammatory factors were measured. The serum activin A and GDF15 in cachectic Cre+ mice showed almost 100‐fold and 10‐fold increase in comparison to that of age‐matched Cre− mice, respectively (*Figure* 2A). However, inflammatory factors IL‐6, IL‐1β, and TNF‐α were not dramatically elevated when compared to the changes in activin A and GDF15 levels (*Figure* 2A). To determine the relative timing and degree of weight loss, we measured serum activin A and GDF15 in correlation with body weight from Cre+ mice. Serum activin A and GDF15 were significantly elevated in Cre+ mice before evidence of body weight loss, suggesting that these two key factors are positively related to CAC progression in Cre+ mice (*Figure* 2B).

### Tumour growth promotes cachectic status through the atrophy of skeletal muscle in Cre+ mice

**Figure 3:**
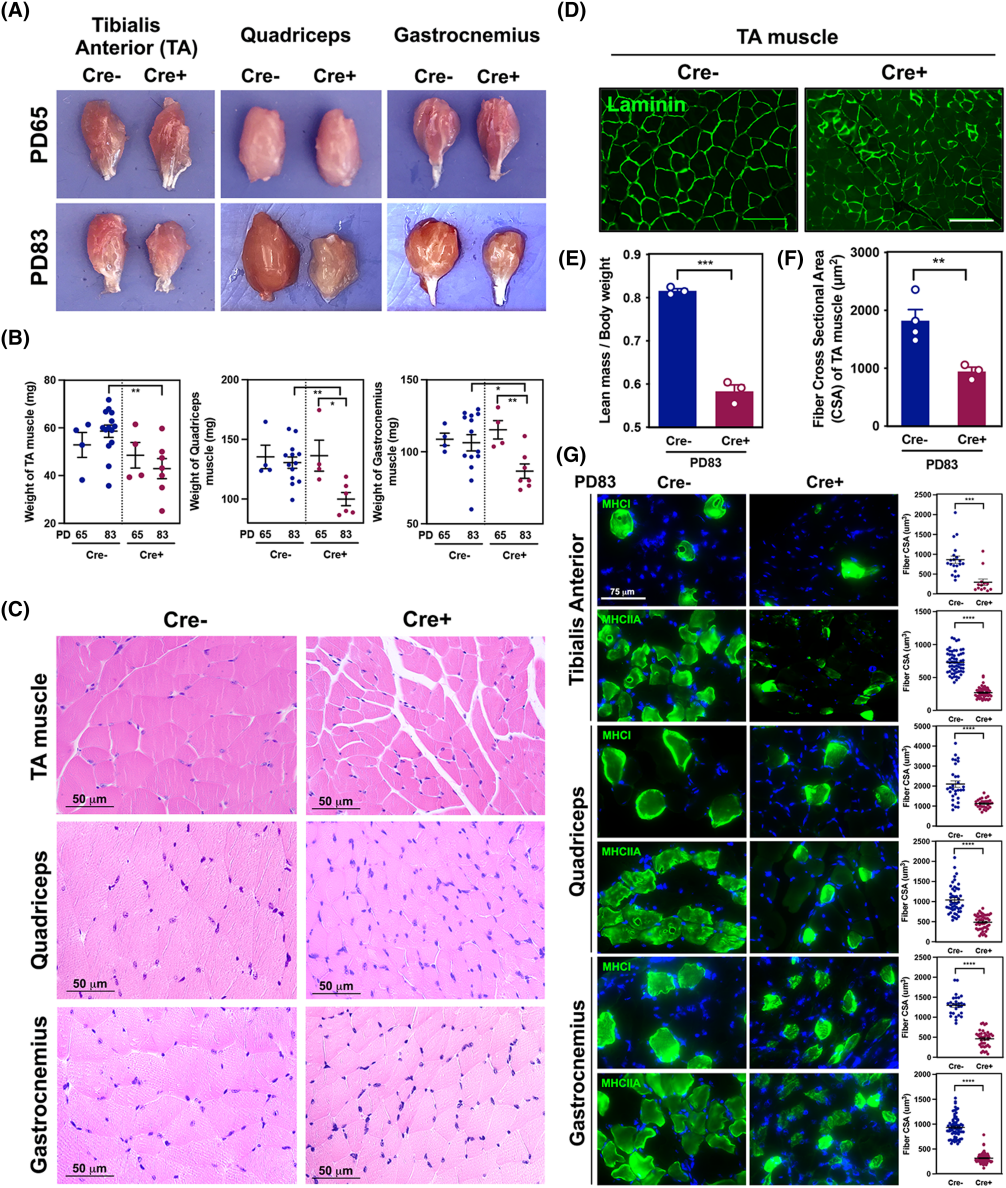
Cancer‐associated cachexia (CAC) causes marked muscle depletion. (*A*) Gross morphology of skeletal muscle (TA, quadriceps, and gastrocnemius muscles). (*B*) Muscle weights in Cre− (*n* = 4–14) and Cre+ (*n* = 4–7) mice on PD65 and PD83. (*C*) Histological images of skeletal muscle from Cre− compared with Cre+ mice. Scale bar = 50 μm. (*D*) Immunofluorescence assay of laminin expression in cross‐sections of TA muscle. Scale bar = 20 μm. (*E*) The ratio of lean mass to initial bodyweight (*n* = 3/ea) using EchoMRI. (*F*) The cross‐sectional area of myotubes in TA muscle from Cre− (*n* = 4) and Cre+ mice (*n* = 3). (*G*) Immunofluorescence assay (left) and CSA measurement (right) of MHCI and MHCIIA expression in TA, quadriceps, and gastrocnemius muscles. Scale bar = 75 μm.

Atrophy of skeletal muscle tissues was visually observed in TA, quadriceps, and gastrocnemius muscle of Cre+ mice at PD83 (*Figures* 3A and S3A), which was supported by significant mass reductions (*Figure* 3B). H&E‐staining revealed smaller skeletal myofibres in Cre+ mice when compared to those in Cre− mice at PD83 (*Figure* 3C). Immunostaining of the TA muscles with laminin presented significantly smaller skeletal myofibres in Cre+ mice compared with Cre− mice (*Figure* 3D). Lean mass in Cre+ mice compared with age‐matched Cre− mice at PD83 was quantitatively reduced (*Figure* 3E). Accordingly, the measurement of myofibre CSA also quantitatively confirmed significant atrophy of muscle fibres in Cre+ mice (*Figure* 3F). Meanwhile, the CSA of both MHCI and MHCIIA‐positive fibres were significantly reduced in TA, quadriceps, and gastrocnemius muscles of Cre+ mice in comparison to Cre− controls (*Figure* 3G).

**Figure 4:**
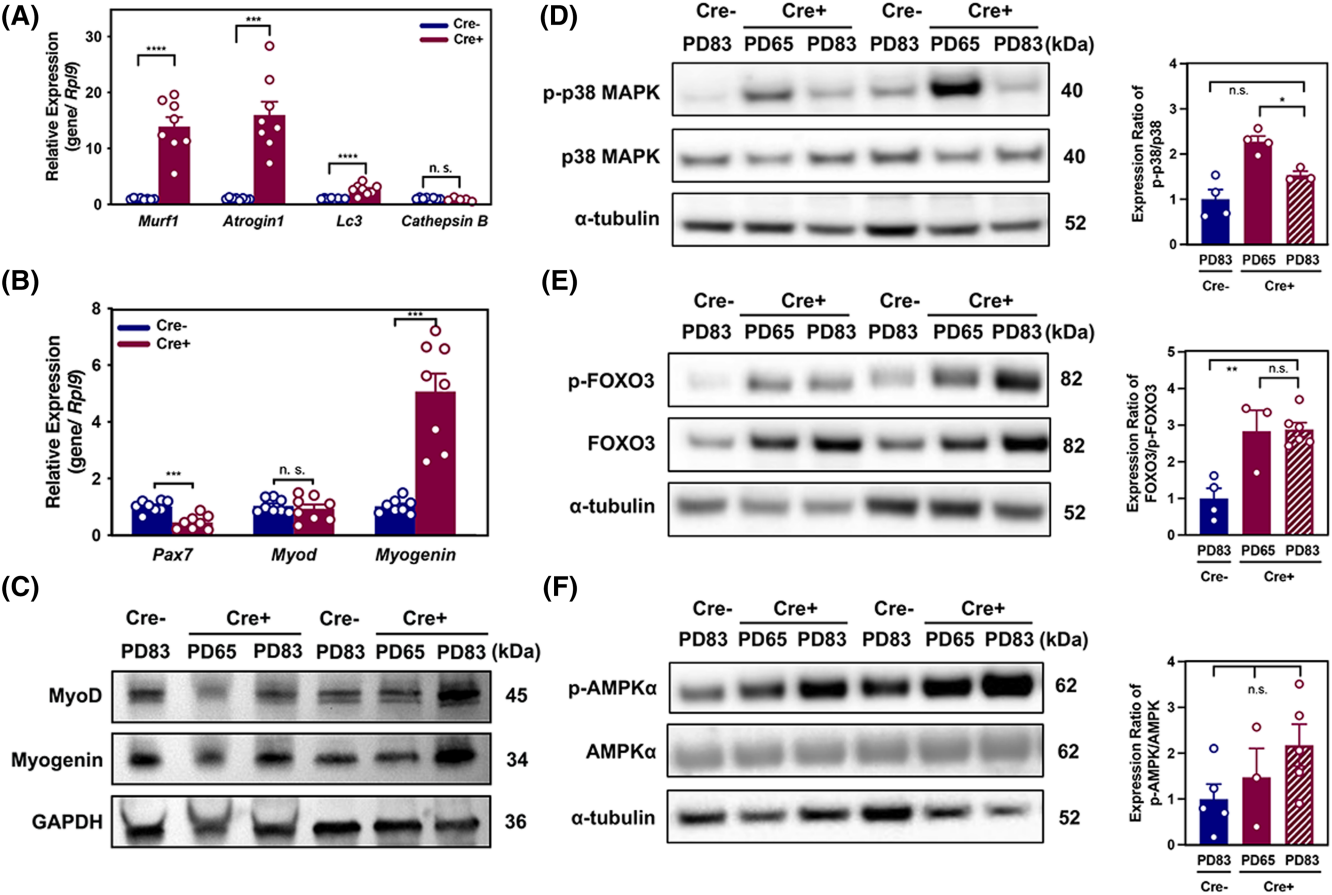
Progression of cancer cachexia is accompanied by changes in the expression of key regulators of muscle function. (*A* and *B*) Real‐time PCR analysis of *Murf1*, *Atrogin1*, *Lc3*, *Cathepsin B*, *Pax7*, *Myod1*, and *Myogenin* in TA muscle from Cre− (*n* = 8) and Cre+ mice (*n* = 8) on PD83. (*C*) Immunoblots of MyoD, Myogenin, and GAPDH in TA muscle lysates (*n* = 3 per group). (*D*) Immunoblots of p‐p38 MAPK, total p38 MAPK, and α‐tubulin in TA muscle lysates with quantification of expression (*n* = 3 per group). (*E*) Immunoblots of p‐FOXO3, total FOXO3, and α‐tubulin in TA muscle lysates with quantification expression (*n* = 3 per group). (*F*) Immunoblots of AMPKα, p‐AMPKα, and α‐tubulin in TA muscle lysates with quantification of expression (*n* = 3 per group).

Given that skeletal muscle wasting is a hallmark of CAC, we measured gene transcripts for muscle‐specific E3 ligases *Murf‐1*, *Atrogin‐1*, and the lysosomal proteolytic factor *Lc3*. These were significantly enriched in the TA muscle from cachectic Cre+ mice, while *Cathepsin B* mRNA levels were not significantly altered compared to Cre− mice (*Figures* 4A, S3B, and S3C). Correspondingly, the expression of *Pax7* genes known to be involved in skeletal muscle regeneration was significantly lower in Cre+ mice in comparison to that of Cre− mice at PD83 (*Figure* 4B), while the expression of PAX7 was below threshold of detection in skeletal muscle tissues. Interestingly, the expression of the myoblast differentiation factor *Myogenin* was significantly higher in the TA muscle from Cre+ mice when compared with Cre− controls although the expression of myoblast proliferation factor *Myod1* was no different (*Figure* 4B). MyoD and myogenin expression were highly increased in the TA muscle at PD83, further confirming that muscle regeneration signalling was intact (*Figure* 4C), while ubiquitin‐proteasome signalling and muscle autophagy were activated in Cre+ mice. However, MyoD expression was reduced during cachexia progression while Myogenin expression was comparable to Cre− control mice in quadriceps and gastrocnemius muscles of Cre+ mice (*Figure* S4A and S4B).

### AMPKα and FOXO3α signalling are up‐regulated in skeletal muscle of cachectic mice

To examine the mechanism of muscle atrophy in Cre+ mice, we investigated the p38 mitogen‐activated protein kinase (MAPK) pathway which is related to activin A stimulation and induces E3 ubiquitin ligases in skeletal muscle.^25^ Phospho‐p38 MAPK/p38 MAPK highly increased in the TA muscle at PD65 but decreased to baseline at PD83 in Cre+ mice (*Figure* 4D). As the expression of *FoxO3* in muscle is strongly correlated with the degree of weight loss, the ratio of FOXO3/phospho‐FOXO3 was up‐regulated in TA muscle from Cre+ mice when compared with age‐matched Cre− mice (*Figures* 4E and S3D). Another pathway related to muscle protein degradation was adenosine monophosphate‐activated protein kinase (AMPK) signalling, which stimulates muscle protein degradation by increasing FOXO3 transcription, thereby stimulating the expression of *Atrogin‐1* and *Murf‐1*.^26^, ^27^ Accordingly, the ratio of phospho‐AMPK/AMPK was significantly up‐regulated in Cre+ mice at PD83, implying that an increase in energy stress in TA muscle as tumour growth progressed (*Figures* 4F).

### Cancer‐associated cachexia induces loss of adipose tissue

**Figure 5:**
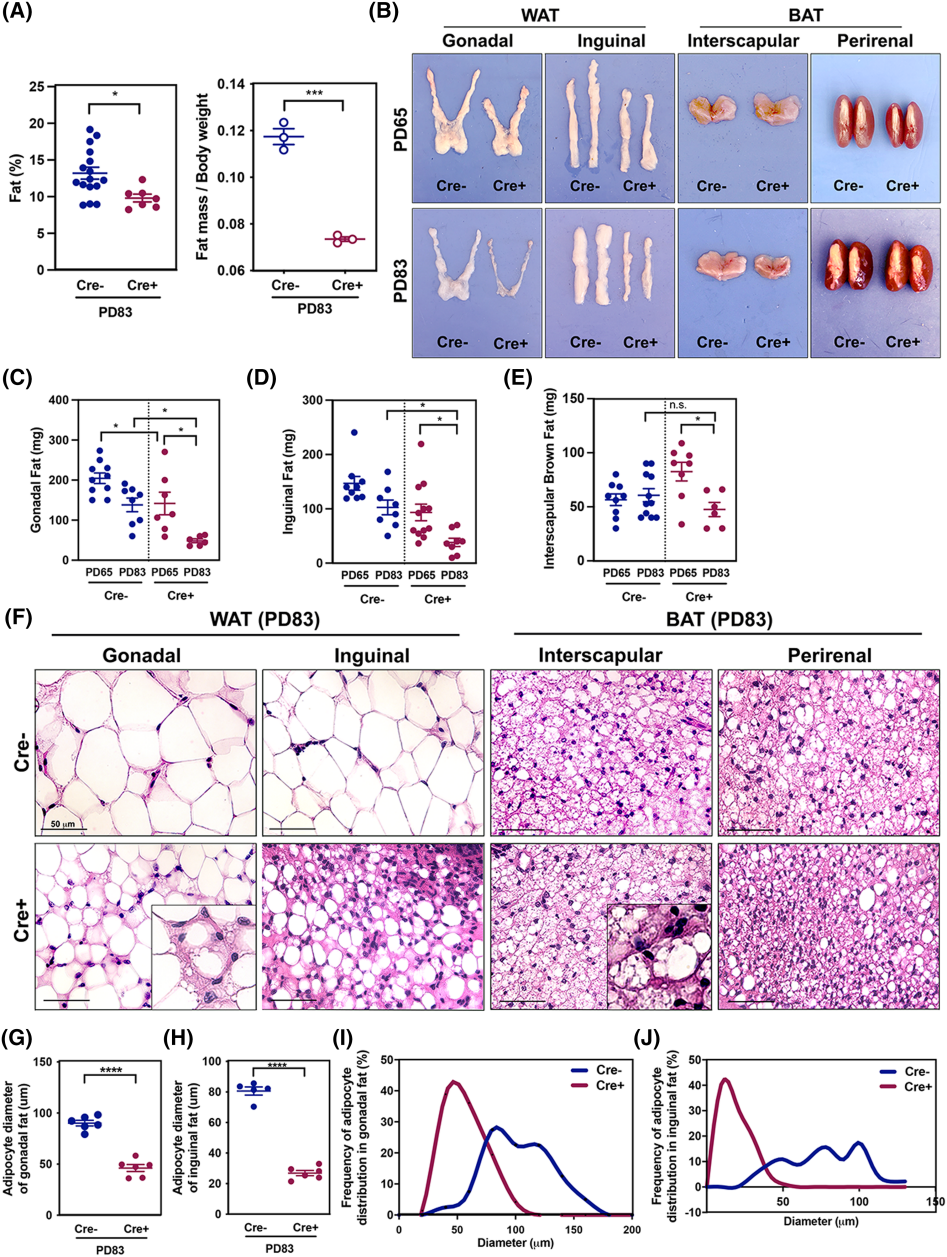
Cancer‐associated cachexia (CAC) drives adipose tissue atrophy. (*A*) Fat tissue percentage of Cre− (*n* = 16) and Cre+ mice (*n* = 7) measured by dual X‐ray absorptiometry scan (left). The ratio of fat mass to initial body weight in Cre− (*n* = 3) and Cre+ mice (*n* = 3) using EchoMRI (right). (*B*) Gross morphology of WAT and BAT. (*C–E*) Weight of adipose tissue of Cre− and Cre+ mice (*n* = 6–11). (*F*) Histological images of adipose tissue. (*G* and *H*) Adipocyte diameter analysis of gonadal (*n* = 6) and inguinal fat (*n* = 6). Each dot represents the average diameter of over 300 adipocytes/one mouse. (*I* and *J*) Frequency distribution of adipocyte diameter in gonadal (*n* = 6) and inguinal (*n* = 6).

To examine the progression of adipose tissue wasting in Cre+ mice, total fat (%) as well as fat mass were measured in Cre+ mice at PD83 (*Figure* 5A). The mean value of fat per cent in Cre− mice was 13.2% in Cre− and 9.8% in Cre+ mice at PD83, showing the average 3.4% of fat loss. Fat mass/body weight also decreased by 62% in Cre+ mice compared with Cre− controls as tumour grew (*Figure* 5A). The gross morphology of adipose tissue at PD65 and PD83 indicated fat loss in WAT and BAT (*Figure* 5B). In particular, the gonadal fat pad, a visceral fat responsible for energy storage, was dramatically reduced (*Figure* 5B). Mass of inguinal WAT and interscapular BAT also significantly decreased during the progression of CAC (*Figure* 5C–5E). Histological analysis showed that the size of adipocytes dramatically decreased in both gonadal and inguinal WAT, while there were no significant changes in BAT (*Figure* 5F). Interestingly, multilocular lipid droplets emerged in both gonadal and inguinal WAT of Cre+ mice (*Figure* 5F). Analyses of both gonadal and inguinal fat pads at PD83 presented significantly reduced diameter of adipocytes in Cre+ mice in a cachectic state (*Figure* 5G and 5H). The skewed frequency distribution in favour of smaller size adipocytes revealed the gross reduction of adipocyte diameter in WAT of cachectic mice (*Figure* 5I and 5J). Taken together, these data confirmed atrophy of adipose tissue during CAC progression in Cre+ mice.

### Cancer cachexia induced adipose tissue remodelling through browning and fibrosis

**Figure 6:**
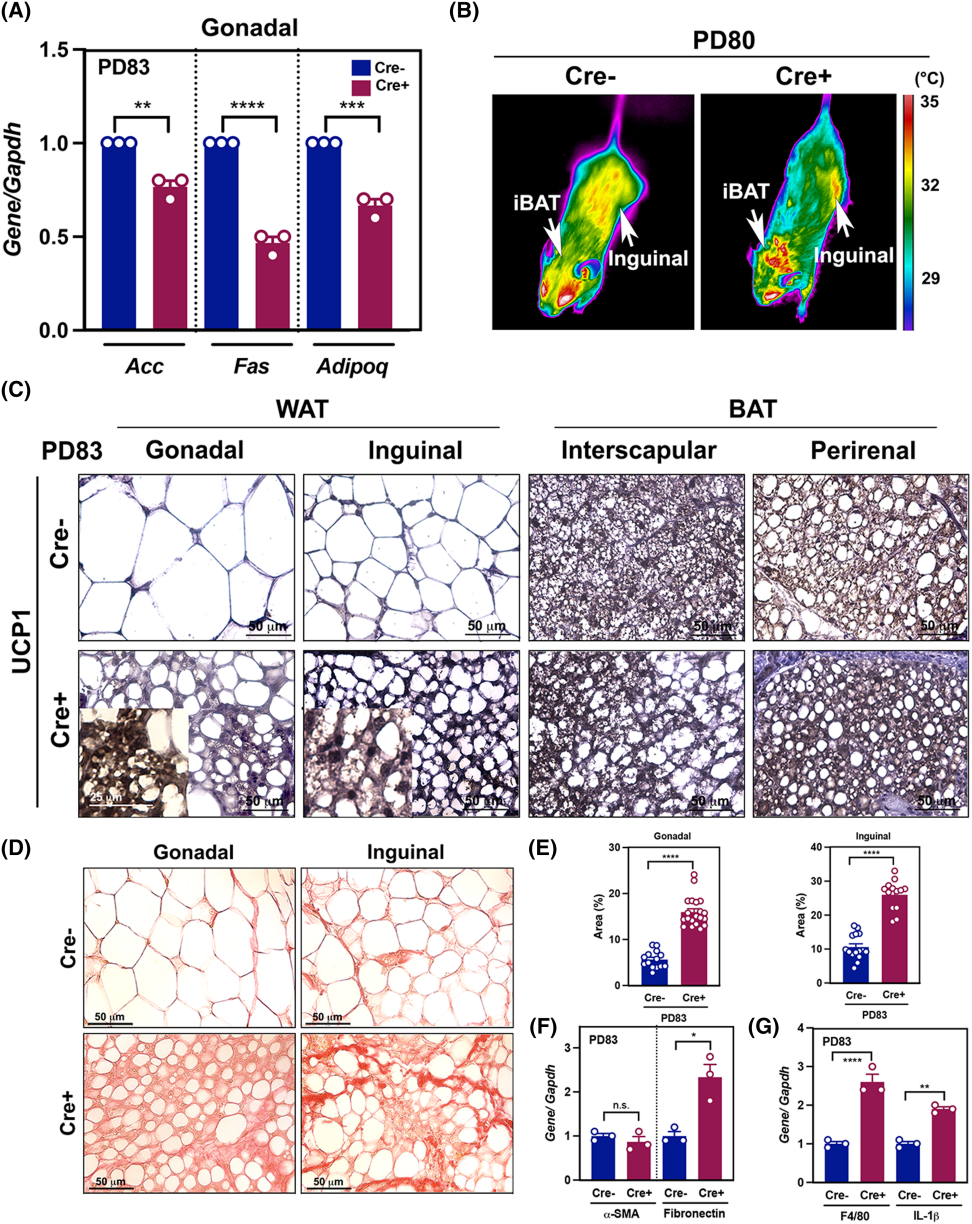
Browning and fibrosis of White adipose tissue (WAT) occur during CAC progression. (*A*) Real‐time PCR (RT‐PCR) analysis of *Acc*, *Fas*, and *Adipoq* in gonadal adipose tissue from Cre− and Cre+ mice (*n* = 3 per group). (*B*) Heat release captured by infrared camera of Cre− and Cre+ mice. (*C*) DAB staining with UCP1 in WAT and BAT from Cre− and Cre+ mice; insets, the expression of UCP1 with high magnification. Scale bar = 20 μm. (*D*) Picro‐sirius red staining of adipose tissue on PD83. (*E*) Area percentage of red pixels indicating collagen I/III deposition in gonadal and inguinal adipose tissue from Cre− and Cre+ mice (*n* = 4 per group) on PD83. (*F* and *G*) RT‐PCR analysis of *α‐SMA*, *fibronectin*, *Adipoq*, *F4/80*, and *IL‐1β* in gonadal adipose tissue from Cre− and Cre+ mice (*n* = 3 per group) on PD83.

To investigate the mechanisms of fat loss in response to CAC, we tested lipogenesis‐related genes, including *Acc* (Acetyl‐CoA carboxylase), *Fas* (fatty acid synthetase), and *Adipoq* (Adiponectin). Analyses of gonadal fat revealed a significant reduction in the expression *Acc* and *Fas*, key regulators of fatty acid synthesis, indicating suppressed *de novo* fatty acid synthesis in cachectic mice (*Figure* 6A). The expression level of *Adipoq*, an adipokine regulating glucose and lipid metabolism, was significantly decreased in Cre+ mice (*Figure* 6A). As known that WAT burns fat (or triglyceride) through increased thermogenesis via browning during CAC, Cre+ mice exhibited an increase in heat release in interscapular BAT and inguinal fat of cachectic mice (*Figure* 6B). Accordingly, the expression of browning specific marker, UCP1, was highly expressed in both inguinal and gonadal WAT of Cre+ mice (*Figure* 6C). Remarkably, WAT from Cre+ mice revealed fibrotic deposition during adipose tissue wasting, as supported by Picro‐sirius red staining (*Figure* 6D) with an increase in area percentage of red pixels in both WAT of Cre+ mice (*Figure* 6E). Real‐time quantitative PCR (RT‐qPCR) data supported that the fibrotic adipose tissue has high levels of fibronectin (*Fn1*) rather than alpha‐smooth muscle actin (*Acta2*) (*Figure* 6F). Up‐regulation of macrophage marker F4/80 (*Adgre1*/*Emr1*) and cytokine *Il1b* in gonadal WAT of cachectic mice was observed (*Figure* 6G).

## Discussion

Cachexia frequently occurs in patients with advanced serous ovarian cancer (OC) and associated with adverse clinical outcomes and reduced survival rate.^1^, ^28^, ^29^ The prevalence of sarcopenia in ovarian cancer cases is up to 54% as reported by meta‐analysis studies.^29^ Furthermore, skeletal muscle index and radiodensity were significantly reduced during ovarian cancer treatment and independent of body mass index change.^28^ The development of ovarian cancer in a xenografted mouse model also demonstrated loss of muscle and bone mass.^30^ The weak body condition of OC patients hinders disease treatment, contributing to morbidity and mortality. In this study, we investigated the progression of spontaneously developed CAC in our ovarian tumour mouse model. We found that our mouse model not only presents ovarian tumours with 100% penetrance but also develops weight loss, muscle atrophy, and adipose tissue wasting in addition to an increase of similar plasma cytokine profile and prominent pathophysiological symptoms as CAC patients. At present, limited animal models of CAC are available for preclinical studies and are only relevant for certain subsets of human conditions.^31^, ^32^ Therefore, there is an unmet need to establish a mouse model that recapitulates human cachexia symptoms and possesses a timely development of CAC to aid in the study of underlying mechanisms of CAC and the development of therapeutics against CAC. In our mouse model, the timing for the initiation of weight loss, the survival, and the changes in body condition are predictable with cachectic symptoms, implying that this is a feasible model to investigate the onset of CAC, the underlying mechanisms of tissue wasting, and metabolic changes, and development of therapeutic agents. Hence, our unique mouse model is a valuable fit for the criteria required for preclinical studies (Table 1).

**Table 1:** Comparison of symptoms between CAC patients and PIK3CA* (Cre+) mouse model

		Cancer cachexia patient				PIK3CA* mouse	
Body weight loss		✓				✓	
Catabolic factors		• Activins • Myostatin • TGFβ	• Serotonin • Parathyroid hormone‐related protein (PTHrP) • Adrenomedullin, miR‐21, HSP70, HSP90			• Activin A ↑ • Myostatin ↑	
Pro‐inflammatory mediators		• IL‐1α • IL‐1β • IL‐6 • IFNγ	• TNF • IL‐11 • IL‐17 • LIF	• GDF15 • TWEAK • TRAF6 • Oncostatin M	• TNFRSF12A • PGE2	• IL‐6 ↑ • GDF15 ↑ • TNFα	
Target Organs	Skeletal muscle	• Myogenesis • *MYOGENIN* • *MYOD1* • *MYF5*		• Proteolysis *• MURF1* *• ATROGIN1* *• NEDD4*		• *Murf1 ↑* *• Atrogin1* ↑ • *Lc3 ↑* *• Pax7* ↑	• Myogenin ↑ • P38 ↑ • FOXO3 ↑ • AMPK ↑
	White adipose tissue	• Adipose tissue wasting • *FAS* • *ADIPOQ* • *ACC*		• Lipolysis • *ATGL* • *HSL*	• Browning • *UCP1* • *PRDM16* *• Cidea*	*• Fas ↓* • *Acc*↓	• *Adipoq* ↓ • UCP1 ↑

Bodyweight loss, pro‐inflammatory mediators, and changes in muscle and white adipose tissue are compared

Importantly, we demonstrated a positive correlation between the increase in serum levels of activin A and GDF15 and progressive weight loss. The cytokine profile of Cre+ mice showed gradual increase in activin A and GDF15 before the initiation of weight loss and consistently elevated serum levels of activin A and GDF15 with progressive weight loss; this pattern is analogous with previously reported cytokine screening results from CAC patients.^20^, ^33^ The elevated serum level of activin A is a result of activin A synthesis and secretion by the fast‐growing granulosa cell tumours, as shown in a previous study of this mouse model.^34^ Because GDF15 signalling has been linked to anorexia through interaction with GDNF family receptor α‐like (GFRAL) in the brain^35^ and has been reported as a factor of CAC, additional studies of GDF15 will be necessary to further understand the progression of CAC using this mouse model.

Muscle wasting and adipose tissue loss are the major hallmarks of CAC syndrome that seriously hamper patients' quality of life. In our CAC model, we observed significant muscle wasting as tumour grew. We detected increased expression of molecules associated with the E3 ubiquitin‐ligase system and the lysosomal proteolytic system. Similar expression patterns have been reported in other CAC mouse models, such as the transgenic inhibin‐α knockout mouse model and syngeneic cancer graft models with C26 colon carcinoma, Lewis lung carcinoma (LLC), and Yoshida AH‐130 hepatoma.^5^, ^36^ Transcription of *myogenin*, which is activated by p‐AKT in skeletal muscled,^37^ was also elevated. The expression of myoblast proliferation factor *Myod1* was unchanged in TA muscle of cachectic Cre+ mice. These data suggest that our cachexia mouse model has relatively normal muscle regeneration activity, including myoblast proliferation and differentiation, as a compensatory response to the acute loss of muscle fibres. These results agree with previous findings that inhibition of protein synthesis was not sufficient to induce muscle wasting in mice and humans.^38^ However, we cannot exclude the contribution of defects in muscle satellite cell activation to muscle wasting in our CAC model, as satellite cell marker *Pax7* was downregulated, which may indicate depletion of the muscle satellite cell reserve.^39^

Adipose tissue atrophy is another driver of weight loss during CAC progression. It has been known that browning of WAT represents an early and systemic event in cachexia pathophysiology and contributes to increased thermogenesis. The high prevalence of BAT (80%) in CAC patients was observed and elevated BAT volume was associated with an increased possibility of tumour recurrence and tumour‐associated mortality.^40^, ^41^ The browning process of WAT is reflected by the increase in the number of beige adipocytes and the expression of UCP1.^12^, ^14^ WAT browning is detrimental as it accelerates energy expenditure and cachexia progression.^10^ Unexpectedly, UCP1 appears to be up‐regulated in visceral WAT of our cachectic mouse model, which has not been observed in other studies.^12^ This phenomenon may reflect the involvement of factors secreted by ovarian tumours, such as oestrogen.^34^ These interesting observations should be further investigated to understand the causal factors of UCP1 up‐regulation in adipose tissue and metabolic dysregulation in ovarian cancer. Recently, it has been reported that fat loss was observed in the absence of muscle loss in cachectic patients with pancreatic ductal adenocarcinoma.^42^ Consistently, WAT mass started to decline at PD65 in our mouse model, while skeletal muscle mass displayed no significant atrophy at the same time. We speculate that adipose tissue loss precedes muscle atrophy during CAC progression at early stages. Moreover, adipose tissue developed highly dense, fibrotic collagen depots during CAC progression. Although the increase in the intensity of fibrosis is correlated with adipose tissue loss, our current study could not reveal the origin and role of fibrosis in adipose tissue.

Here, we characterized the progression of spontaneously developing cancer cachexia using a novel transgenic mouse model. This valuable model therefore is ideally suited to track and study the development of cancer cachexia at different stages to better understand the pathophysiological mechanisms of cachexia and to test therapeutics that target key factors.

## Conflict of interest

The authors declare no competing interests.

## Supporting information


**Table S1.** List of primer sequences.
**Table S2.** List of primary and secondary antibodies.

**Figure S1.** Cre + mice develop gross cachectic phenotypes as ovarian tumours grow **(A)** Genetic background of Pik3ca* mice. (**B**) Dorsal images of Cre‐ and Cre + mice. The hunched posture of Cre+ mouse is indicated with an arrow. (**C**) Image of incisors from Cre‐ and Cre+ mice. Elongated incisors of the Cre+ mouse are indicated with arrows. (**D**) MRI image of ovarian tumours (red arrows) in Cre+ and Cre‐ mice. **(E)** Image of the abdomen from Cre+ and Cre‐ mice. Tumour growth and fat loss were shown in Cre+ mice.

**Figure S2.** Cancer cachexia alters organ features and body composition of Cre+ mice **(A)** Images of spleen, pancreas, and liver. (**B**) Histological images of liver and stomach in Cre‐ and Cre+ mice. (**C and D**) Bone area, tissue area, total tissue mass, and soft tissue density of Cre‐ (*n* = 16) and Cre+ (*n* = 7) mice on PD83 using DEXA scan.

**Figure S3.** Body weight loss positively correlates with muscle atrophy **(A)** TA muscle of the hind limbs of Cre‐ and Cre+ mice. (**B**) Correlation between *Atrogin‐1* and *Murf1* mRNA expression in TA muscle and percentage of weight loss from Cre+ mice (*n* = 16). (**C**) qPCR analysis of *Lc3* in skeletal muscle from Cre‐ (*n* = 4) on PD83 and Cre+ mice on PD65 (*n* = 5) and PD83 (*n* = 3). Correlation between the expression of *Lc3* and weight loss in Cre+ mice. (**D**) Correlation between the expression of *Foxo3* and weight loss in Cre+ mice.

**Figure S4.** Expression of muscle regeneration factors alter during CAC progression (A) Immunoblots of MyoD, Myogenin, and GAPDH in gastrocnemius muscle lysates (*n* = 3). (B) Immunoblots of MyoD, Myogenin, and GAPDH in quadriceps muscle lysates (n = 3).
