# Supplementary material for: Development of ovarian tumour causes significant loss of muscle and adipose tissue: a novel mouse model for cancer cachexia study
Source: J Cachexia Sarcopenia Muscle. 2022 Jan 19;13(2):1289–301. doi: 10.1002/jcsm.12864 (PMC8977964; doi:10.1002/jcsm.12864)
Supplement: Supplementary file 1 — Table S1. List of primer sequences. Table S2. List of primary and secondary antibodies. [file JCSM-13-1289-s004.docx]

**Supplemental Material**

**Development of ovarian tumor causes significant loss of muscle and adipose tissue: a novel mouse model for cancer cachexia study**

Yi Luan^1#^, Yaqi Zhang^2#^, **Seok-yeong Yu^1^**, Mikyoung You^3^, Pauline C. Xu^1^, Soonkyu Chung^3^, Takeshi Kurita^4^, Teresa K Woodruff^2^, Jie Zhu^2*^, So-Youn Kim^1*^

**Affiliations:**

^1^Olson Center for Women’s Health, Department of Obstetrics and Gynecology, Fred & Pamela Buffett Cancer Center, College of Medicine, University of Nebraska Medical Center, Omaha, NE

^2^Division of Reproductive Science in Medicine, Department of Obstetrics and Gynecology, Feinberg School of Medicine, Northwestern University, Chicago, IL

^3^Department of Nutrition, School of Public Health and Health Sciences, University of Massachusetts Amherst, Amherst, MA

^4^Department of Cancer Biology & Genetics, The Comprehensive Cancer Center, Ohio State University, Columbus, OH

# Both authors contributed equally to this work

* Co-corresponding authors

**Supplemental Figures**

**Figure S1.**

**Figure S2.**

**Figure S3.**

**Figure S4.**

**Supplemental Tables**

**Table S1.** List of primer sequences

**Table S2.** List of primary and secondary antibodies

**Supplemental Figure Legends**

**Table. S1 List of primer sequences**

| **Gene Symbol** | **Gene Aliase** | **Gene Name** | **Assay ID** | **Species** |
| --- | --- | --- | --- | --- |
| *Trim63* | MuRF1 | Tripartite motif-containing 63 | Mm01185221_m1 | Mouse |
| *Fbxo32* | Atrogin1 | F-box protein 32 | Mm00499523_m1 | Mouse |
| *Map1lc3a* | *Lc3a* | microtubule associated protein 1 light chain 3 alpha | Mm00458724_m1 | Mouse |
| *Ctsb* |  | cathepsin B | Mm00514439_m1 | Mouse |
| *Pax7* |  | paired box 7 | Mm00834082_m1 | Mouse |
| *Myod1* | *MyoD* | myogenic differentiation 1 | Mm00440387_m1 | Mouse |
| *Myog* |  | myogenin | Mm00446194_m1 | Mouse |
| *UCP1* |  | uncoupling protein 1 | Mm01244861_m1 | Mouse |
| *Pparg* |  | peroxisome proliferator activated receptor gamma | Mm00440940_m1 | Mouse |
| *Prdm16* |  | PR domain containing 16 | Mm00712556_m1 | Mouse |

**Table. S2 List of primary antibodies**

| **Protein name** | **Catalog No.** | **Manufacturer** | **Host** | **Dilution** |
| --- | --- | --- | --- | --- |
| **MyoD** | **D7F2** | **DSHB** | **Mouse** | **1:3000** |
| **MYOG/Myogenin** | **LS-C392323** | **LSBio** | **Mouse** | **1:3000** |
| **GAPDH** | **#2118S** | **Cell Signaling Technology** | **Rabbit** | **1:5000** |
| Phospho-p38 MAPK (Thr180/Tyr182) | #9211 | Cell Signaling Technology | Rabbit | 1:1000 |
| p38 MAPK | #9212 | Cell Signaling Technology | Rabbit | 1:1000 |
| Phospho-FoxO1 (Thr24)/FoxO3a (Thr32) | #9464 | Cell Signaling Technology | Rabbit | 1:1000 |
| FoxO3a (75D8) | #2497 | Cell Signaling Technology | Rabbit | 1:1000 |
| Phospho-AMPKα (Thr172) (40H9) | #2535 | Cell Signaling Technology | Rabbit | 1:1000 |
| AMPKα | #2532 | Cell Signaling Technology | Rabbit | 1:1000 |
| Anti-α-Tubulin Antibody, clone DM1A | 05-829 | Millipore Sigma | Mouse | 1:1000 |

List of secondary antibodies

| **Antibody** | **Catalog No.** | **Conjugation** | **Company** | **Dilution** |
| --- | --- | --- | --- | --- |
| Anti-rabbit IgG | 7074 | HRP | Cell Signaling Technology | 1:2000 |
| Anti-mouse IgG | 7076 | HRP | Cell Signaling Technology | 1:2000 |
| Anti-rabbit IgG | BA-1000-1.5 | Biotinylated | Vector Laboratories | 1:400 |
| Anti-mouse IgG | BA-2000-1.5 | Biotinylated | Vector Laboratories | 1:400 |
