# Supplementary figures and images for: Development of ovarian tumour causes significant loss of muscle and adipose tissue: a novel mouse model for cancer cachexia study
Source: J Cachexia Sarcopenia Muscle. 2022 Jan 19;13(2):1289–301. doi: 10.1002/jcsm.12864 (PMC8977964; doi:10.1002/jcsm.12864)

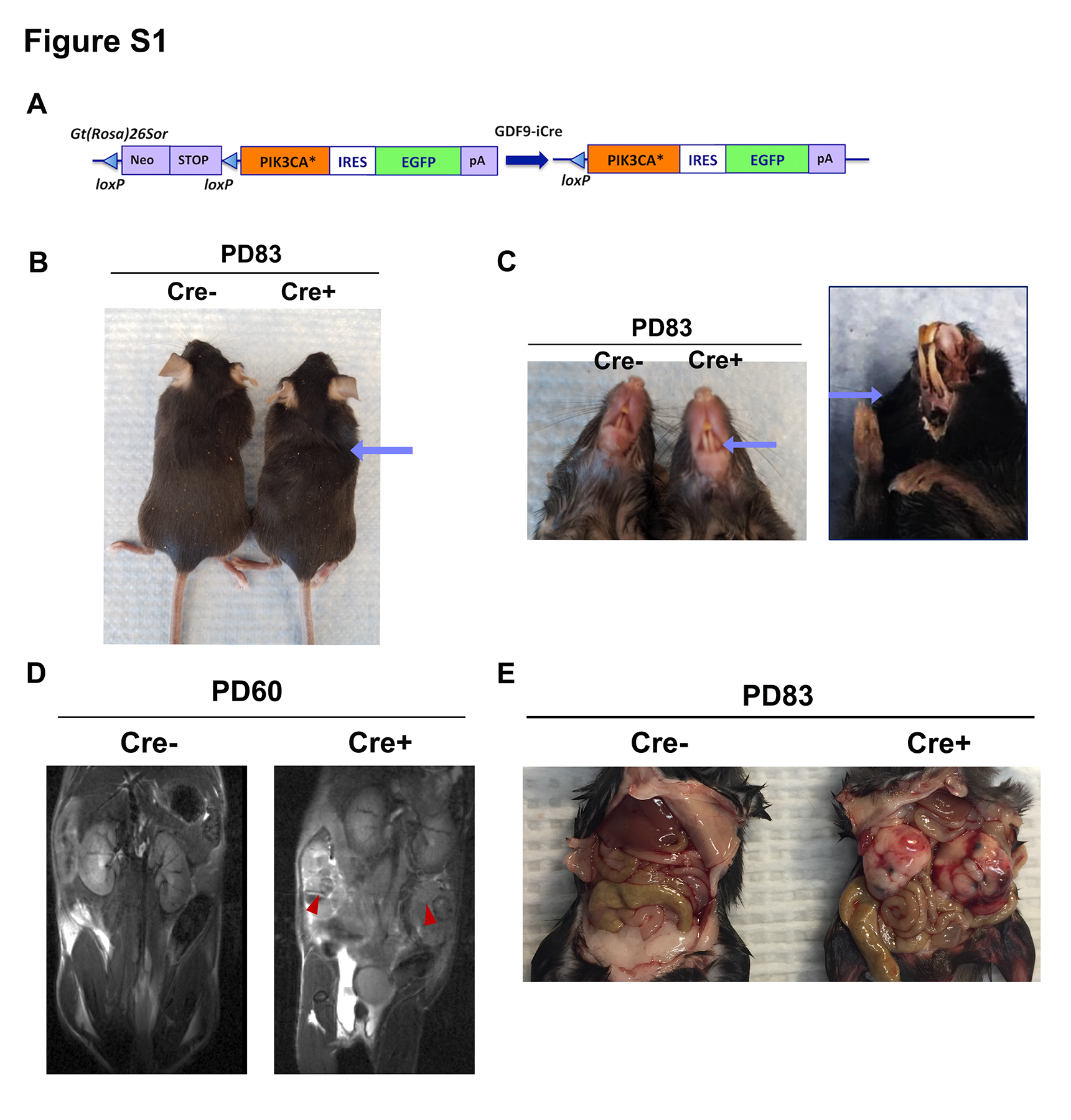

Supplement: Supplementary file 2 — Figure S1. Cre + mice develop gross cachectic phenotypes as ovarian tumours grow (A) Genetic background of Pik3ca* mice. (B) Dorsal images of Cre‐ and Cre + mice. The hunched posture of Cre+ mouse is indicated with an arrow. (C) Image of incisors from Cre‐ and Cre+ mice. Elongated incisors of the Cre+ mouse are indicated with arrows. (D) MRI image of ovarian tumours (red arrows) in Cre+ and Cre‐ mice. (E) Image of the abdomen from Cre+ and Cre‐ mice. Tumour growth and fat loss were shown in Cre+ mice. [file JCSM-13-1289-s005.tif]

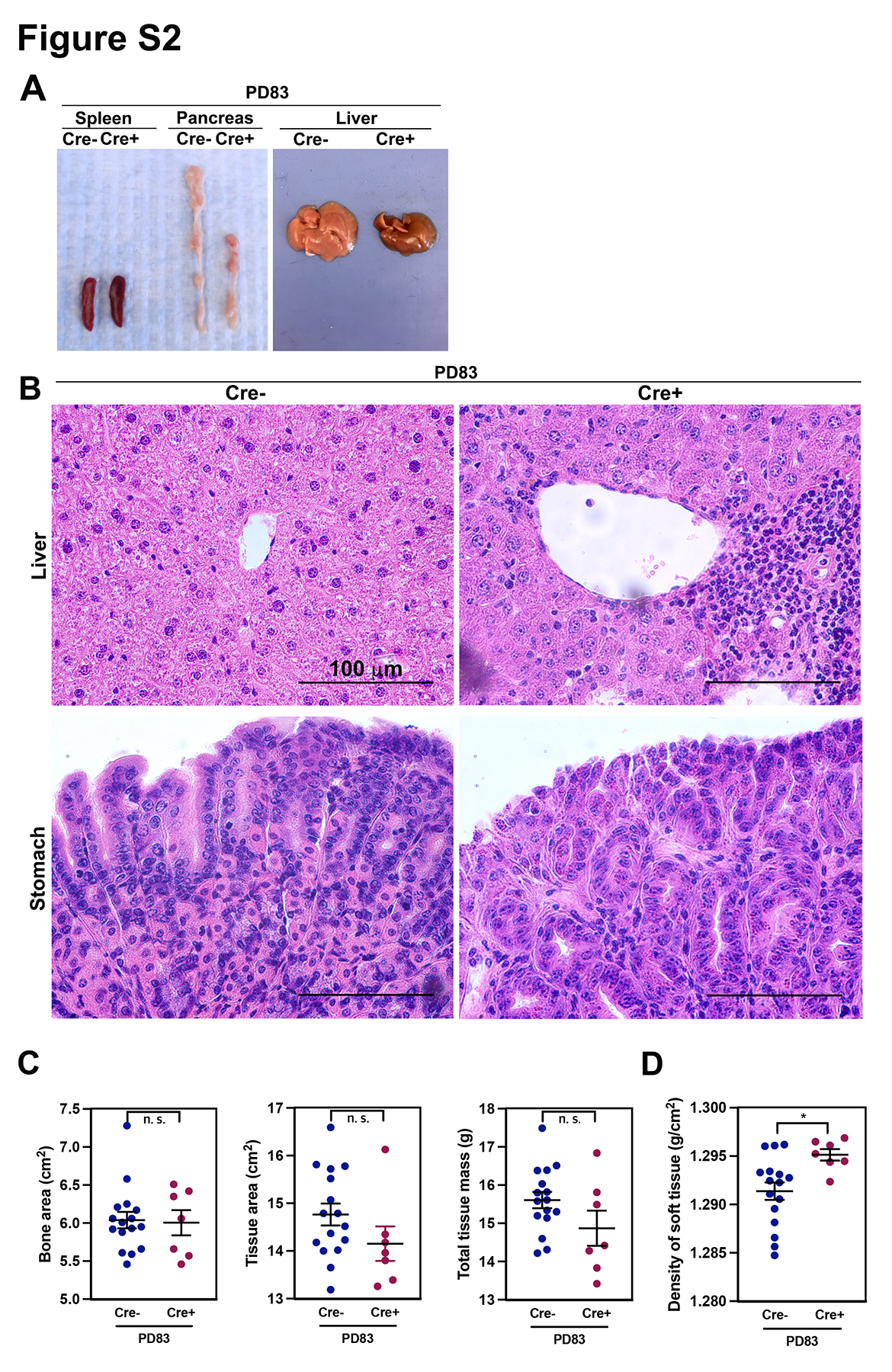

Supplement: Supplementary file 3 — Figure S2. Cancer cachexia alters organ features and body composition of Cre+ mice (A) Images of spleen, pancreas, and liver. (B) Histological images of liver and stomach in Cre‐ and Cre+ mice. (C and D) Bone area, tissue area, total tissue mass, and soft tissue density of Cre‐ (n = 16) and Cre+ (n = 7) mice on PD83 using DEXA scan. [file JCSM-13-1289-s003.tif]

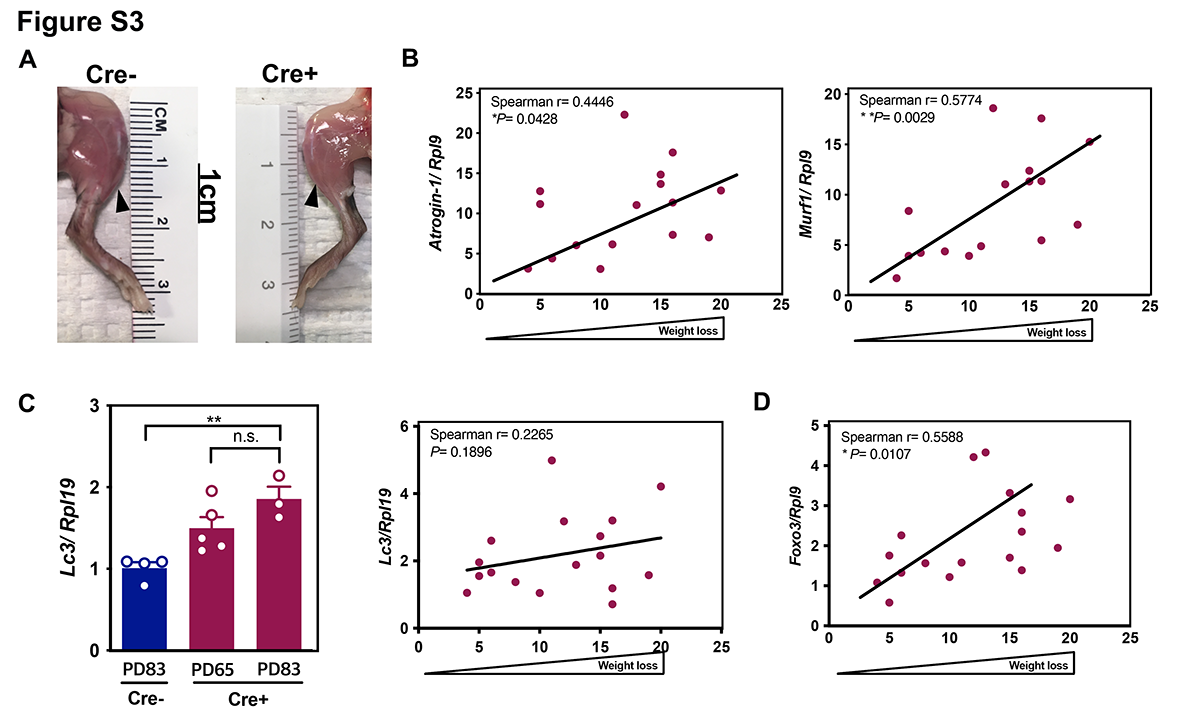

Supplement: Supplementary file 4 — Figure S3. Body weight loss positively correlates with muscle atrophy (A) TA muscle of the hind limbs of Cre‐ and Cre+ mice. (B) Correlation between Atrogin‐1 and Murf1 mRNA expression in TA muscle and percentage of weight loss from Cre+ mice (n = 16). (C) qPCR analysis of Lc3 in skeletal muscle from Cre‐ (n = 4) on PD83 and Cre+ mice on PD65 (n = 5) and PD83 (n = 3). Correlation between the expression of Lc3 and weight loss in Cre+ mice. (D) Correlation between the expression of Foxo3 and weight loss in Cre+ mice. [file JCSM-13-1289-s002.tif]

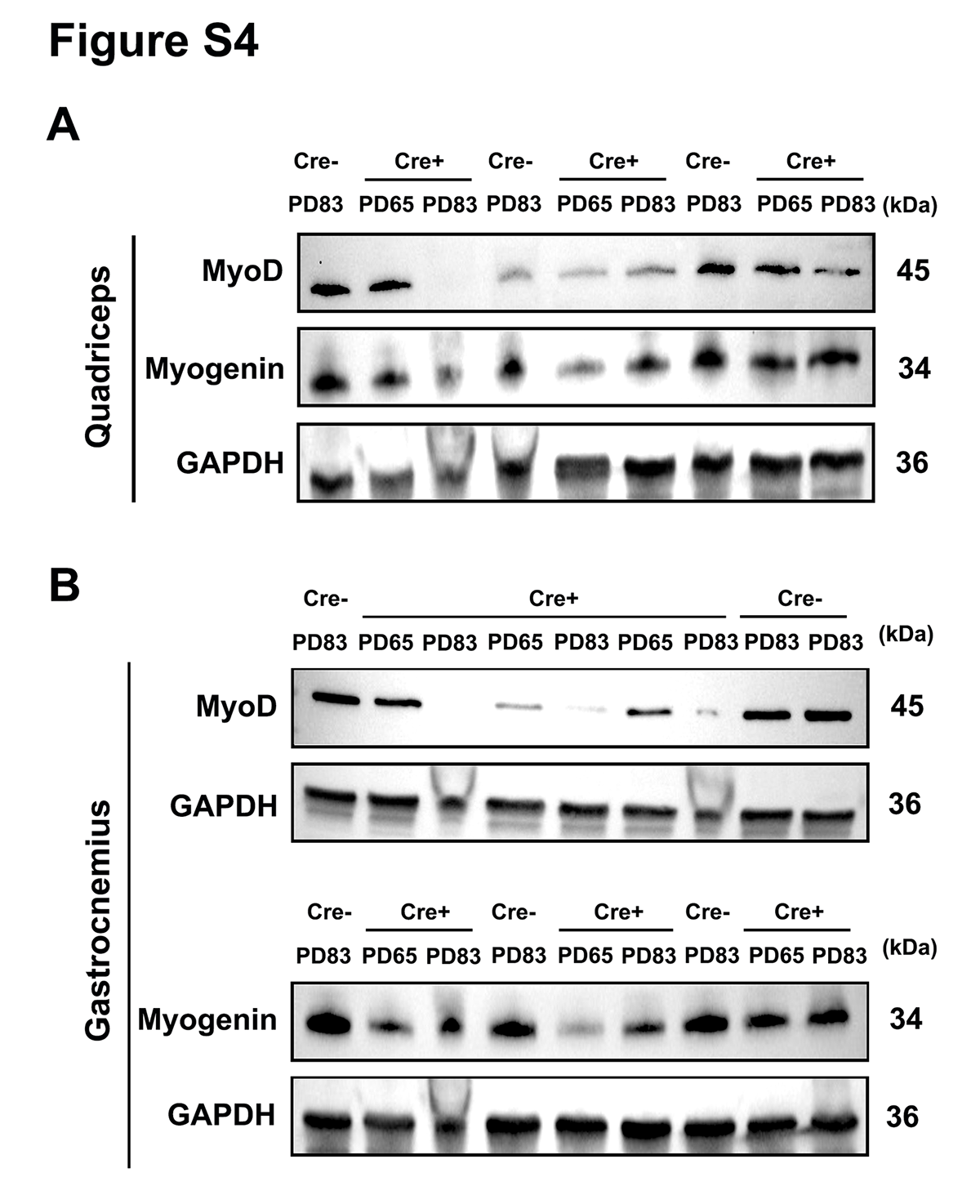

Supplement: Supplementary file 5 — Figure S4. Expression of muscle regeneration factors alter during CAC progression (A) Immunoblots of MyoD, Myogenin, and GAPDH in gastrocnemius muscle lysates (n = 3). (B) Immunoblots of MyoD, Myogenin, and GAPDH in quadriceps muscle lysates (n = 3). [file JCSM-13-1289-s001.tif]
